# Municipal Solid Waste Incineration (MSWI) Ashes as Construction Materials—A Review

**DOI:** 10.3390/ma13143143

**Published:** 2020-07-15

**Authors:** Byoung Hooi Cho, Boo Hyun Nam, Jinwoo An, Heejung Youn

**Affiliations:** 1 Department of Civil, Environmental and Construction Engineering, University of Central Florida, Orlando, FL 32816, USA; byoungcho@ucf.edu; 2 Department of Engineering, University of Mount Union, Alliance, OH 44601, USA; anji@mountunion.edu; 3 Department of Civil Engineering, Hongik University, Seoul 04066, Korea

**Keywords:** municipal solid waste incineration (MSWI) ash, bottom ash and fly ash, cement and concrete, hot-mix asphalt, leaching properties, material characterization

## Abstract

Over the past decades, extensive studies on municipal solid waste incineration (MSWI) ashes have been performed to develop more effective recycling and waste management programs. Despite the large amount of research activities and the resulting improvements to MSWI ashes, the recycling programs for MSWI ashes are limited. For instance, although the U.S. generates more MSWI ashes than any other country in the world, its reuse/recycle programs are limited; bottom ash and fly ash are combined and disposed of in landfills. Reuse of MSWI ashes in the construction sectors (i.e., geomaterials, asphalt paving, and concrete products) as replacements for raw materials is one of most promising options because of the large consumption and relatively lenient environmental criteria. The main objective of this study was to comprehensively review MSWI ashes with regard to specific engineering properties and their performance as construction materials. The focus was on (1) the current practices of MSWI ash management (in particular, a comparison between European countries and the U.S.), (2) the engineering properties and performance of ashes when they are used as substitutes of construction materials and for field applications, and (3) the environmental properties and criteria for the use of MSWI ashes. Overall, the asphalt and concrete applications are the most promising, from both the mechanical and leachate viewpoints. However, cons were also observed: high absorption of MSWI ash requires a high asphalt binder content in hot-mix asphalt, and metallic elements in the ash may generate H_2_ gas in the high-pH environment of the concrete. These side effects can be predicted via material characterization (i.e., chemical and physical), and accordingly, proper treatment and/or modified mix proportioning can be performed prior to use.

## 1. Introduction

The incineration of municipal solid waste (MSW) with energy recovery and the management of MSW incineration (MSWI) ashes are attracting increasing attention around the world. Many countries have pursued the beneficial utilization of MSWI ashes by executing strategic management plans and regulations [1,2,3,4,5,6,7]. For example, several European countries utilize MSWI bottom ash (BA) as sustainable construction materials to realize economic and environmental benefits, in accordance with environmental criteria set by their strategic regulations [2,3,4,8]. The U.S. produces more MSW than any other country in the world; however, the recycling rate is low [9]. The total MSW generation in the U.S. has increased by 65% since 1980 to the current level of 250 million tons per year, with 53.6% landfilled, 34.7% recycled and composted, and 11.7% incinerated with energy recovery [10]. While a total of 86 MSW incineration plants were operated in 24 states of the U.S. in 2010 [11], the number of MSWI plants was only 75 (in 21 states) in 2018 [12]. The major states that use MSWI plants are Florida, New York, Massachusetts, Minnesota, Connecticut, New Jersey, and Pennsylvania [6,12]. Typical residues produced from these incineration plants include MSWI BA and fly ash (FA), which in most cases are combined to be disposed of in a sanitary landfill in the U.S. [7].

As the volume of waste generation continues to increase, incineration technology that reduces the weight and volume of waste by approximately 60% and 90%, respectively, has been adopted for the management of MSW [2]. The process of MSW incineration is generally divided into three main parts: incineration, energy recovery, and air-pollution control (APC) [13,14,15]. The produced ash is referred to as MSWI ash (different from coal FA, which is a byproduct of pulverized coal combustion in electric power plants). Most modern incinerators are equipped with energy-recovery schemes, which produce Waste-to-Energy ash. Three major classes of technologies are used to combust MSW: mass burn (MB), refuse-derived fuel (RDF), and fluidized-bed combustion (FBC) [2]. Most MSWI facilities are MB plants that burn the MSW as received, after the recovery of metals for recycling. RDF plants facilitate the preprocessing of MSW to remove non-combustibles and to shred the MSW into uniform fuel pellets. FBC plants incinerate RDF in a hot fluidized bed of noncombustible granule-like sand in a furnace.

BA, FA, and APC residue are the main products of MSWI. BA is grate ash discharged from the furnace grate and collected in the water quenching tank. During the process, the BA is combined with grate-shifting (fine particles falling through the furnace) and heat-recovery ash (particulate matter collected from the heat-recovery system). FA refers to fine particles that are carried over the furnace and separated before the injection of sorbents to treat the gaseous effluent. Gas condensates and reaction products are produced by APC devices, such as electrostatic precipitators and scrubbers. APC residue is then produced through the combination of FA, sorbents, gas condensates, and reaction products in APC devices. In the U.S., most MSWI plants combine the BA and FA from APC devices in one stream [3,16], which is referred to as “combined ash,” in contrast to European countries, where ashes are separately managed.

According to literature published in the past two decades, the major concern associated with MSWI is the air pollution caused by dioxin (C_4_H_4_O_2_), furan (C_4_H_4_O), and heavy metals originating from MSW [16,17,18,19,20,21]. The emission was significantly reduced by implementing APC devices to treat the toxic flue gases with sorbents using dry/semi-dry and wet scrubber systems [2,5,16]. The employment of APC devices shifted the concern from air pollution to the leachate from the disposal of MSWI ashes in landfills. It has been reported that RDF processes provide significant control over the release of heavy metals, reducing the amounts of Pb, Cd, and Cr by 52, 73, and 63 wt.%, respectively [22].

Various studies have been performed on MSWI ashes, focusing on either the engineering or environmental aspect with regard to the application fields, either BA or FA with regard to the ash type, and either cementitious or bituminous with regard to the binding materials. Ferreira et al. (2003) studied the metal concentrations in MSWI FA and concluded that the FA contains several heavy metals, such as Zn, Pb, Fe, Mg, Mn, Cr, and Cd, which may significantly influence the sustainability and environmental aspects [17]. Luo et al. (2019) examined the leaching behavior of MSWI ashes and reviewed the treatment methods that have been proposed for reducing the amounts of toxic and harmful elements; however, there was limited information regarding the effectiveness of the treatment methods [23]. Lam et al. (2010) reviewed the characteristics of MSWI, with a focus on the chemical properties of the ashes. Seven application fields for which the ashes have potential were reviewed: cement production, road pavement, glasses and ceramics, agriculture, stabilizing agents, adsorbents, and zeolite production [24]. Siddique (2010) and Atoo et al. (2018) examined the potential of MSWI ash for use in concrete by replacing up to 40 wt.% of the aggregate, but limited information was provided on the mechanical properties and performance of the ash-containing concrete; the results indicated that <15% replacement can be achieved for non-structural concrete works [25,26]. Later, Lynn et al. (2016) investigated the use of MSWI BA as an aggregate in concrete and concluded that appropriate pretreatment allowed it to be used as a fine or coarse aggregate in cement mortar, concrete, and blocks [27]. Additionally, in 2017, they presented an extensive review of the potential of MSWI BA for road applications; MSWI BA has potential not only as an aggregate in cement-bound subbase and roadbase layers but also for bituminous bound layers at a low content [28].

The main objectives of this study were to comprehensively review the potential applications of MSWI BA and FA from a construction material viewpoint, with consideration of environmental regulations, and to encourage the recycling and reuse of the ashes in the construction industry. The literature review focused on the following three areas: (1) the current practices of MSWI ash management in European countries and the U.S., (2) the utilization of MSWI ashes as construction materials (with emphasis on the properties and performance), and (3) the environmental impacts of MSWI ashes and regulations related to their use. Finally, a promising application method and limitations of MSWI ashes for use as construction materials are discussed.

## 2. Management Practices for MSWI Ashes

**Figure 1:**
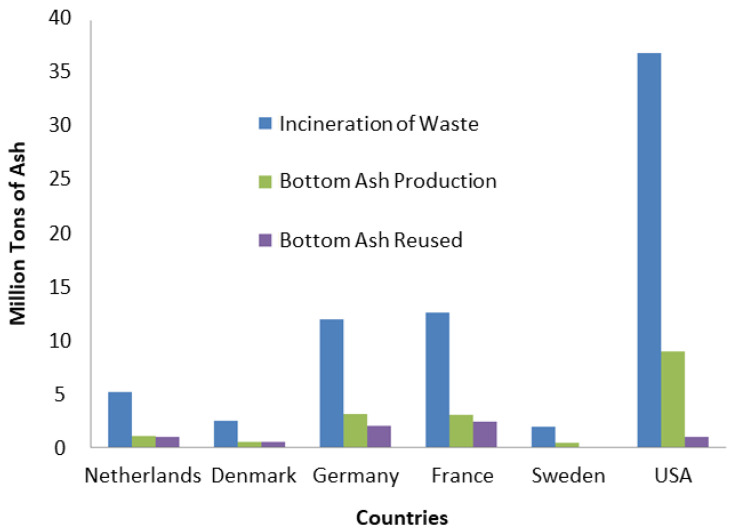
Management practices of different countries for municipal solid waste incineration (MSWI) bottom ash (BA) [31].

The Confederation of European Waste-to-Energy Plants reported that approximately 371 incineration plants treated approximately 85% of the total MSW in Europe from 2001 to 2011 [29]. The number of plants had increased significantly (to 512) as of 2016 [30]. Consequently, the incineration and recycling volumes were increased by 7% and 12%, respectively, and the landfilling volume was reduced by 19% [29]. The renewable-energy contribution is expected to increase to at least 27% by 2030 [30]. Figure 1 shows the management practices of different countries for MSWI BA. As mentioned in Section 1, the U.S. has a very low recycling ratio but generates the largest amount of waste among the world’s countries [9]. Additionally, with regard to the technical aspects, in most European countries, BA, FA, and APC residue are separated, and separate treatment systems are used for environmentally safe landfilling of less useful BA and FA and APC residues to minimize the leachability [3,4,5]. In contrast, in the U.S., most MSWI plants combine BA and FA into one stream and dispose of them in landfills [3,16].

In the U.S., a significant amount (>6.6 million tons) of MSWI ashes is generated annually, but the ashes are hardly utilized for beneficial applications [32,33]. The combined ash (mixed BA and FA) have been mostly disposed of in landfills, and neither federal- nor state-level waste policies address ash reuse [32]. The predominant method for the management of combined ash is disposal in a monofill lined with clay, synthetic liners, or combinations of methods associated with leachate collection and treatment [3]. Currently, in the U.S. there is no recycling of MSWI ash; instead, ash management only involves preprocessing, e.g., the recovery of ferrous metals using magnetic separators and nonferrous metals using an eddy current in facilities with incineration plants [1,3,7]. In contrast, many European countries have reused MSWI ashes [34]. In the Netherlands, approximately 80% of the BA produced is recycled in civil-engineering applications after certain treatment schemes, such as ferrous and nonferrous metal recovery and size reduction [4,35]. The use of BA is encouraged, and it is considered as a special category of materials for embankment fills, road bases, and landfill disposal [4]. Among the European countries, only the Netherlands utilizes—and the use is minimal [3,5]. Approximately 30% of FA and APC residues are used as filler materials in asphalt, as alternatives to limestone [5]. A significant portion of these residues is exported to Germany and used as backfilled material in coal and salt mines [5]. In Denmark, BA is considered to be a suitable gravel substitute as a subbase material when used with an asphalt or concrete cover to avoid direct contact with soil and water [4]. Denmark aimed at recycling 98% of BA into building and road construction and embankment fills after screening, crushing, and ferrous metal recovery [4]. APC residues, including FA and acid cleaning end products, are considered as special hazardous waste for which treatment and landfilling are required [5]. Denmark exports APC residue to Norway for use in neutralizing acid waste and to Germany for use as a backfill in salt mines [5]. In Germany, industry involved in the treatment of BA from MSWI has been attempting to increase the recovery rate and enhance the quality of the aggregate obtained using BA [36]. Consequently, Germany recycles approximately 65% of BA and landfills 28% after the reduction of the salt content via water quenching, followed by ferrous and nonferrous metal recovery and three-month maturation [2,4]. A reduction in the leaching potential makes BA suitable as a road construction or secondary building material [3,8]. The salt in ash and dry scrubber residues is used as backfill in old mines to prevent subsidence [3,5]. A small quantity of APC residue is disposed of in landfills after stabilization [5]. Studies performed in France regarding the use of MSWI FA and BA in the construction industry indicated that the ashes are widely used for the stabilization of industrial waste sludge, road basement materials, and aggregates for concrete [37]. It was reported that France recycles 79% of BA produced in civil construction [4]. BA treatments involve ferrous and nonferrous metal removal, size reduction, and sometimes cement stabilization [4]. APC residue management is mostly accomplished via cement and chemical stabilization using NaHCO_3_ and disposal into landfills designated for hazardous waste [5]. Additionally, thermal treatment is considered as a new option for ash treatment but is not yet common [5]. Lastly, in Sweden, MSWI BA is widely used as a filling material below landfill top covers and as a construction material on a smaller scale after pretreatment via metal recovery and carbonation [38]. FA is disposed of in special lined landfills or cells after treatment [3]. Sweden exports APC residues to Norway for neutralization of acid waste and landfilling after solidification and stabilization [5].

## 3. MSWI Ashes as Construction Materials

### 3.1. Properties of MSWI Ashes

#### 3.1.1. MSWi BA

BA is the major byproduct residue of the MSWI process (85–95 wt.%) and comprises grate ash and sometimes grate-shifting ash. BA is a porous, grayish, and coarse gravel material containing primarily glasses, ceramics, minerals, and ferrous and nonferrous materials, along with small amounts of unburned materials and organic carbon [2,3]. The major compounds are oxides, hydroxides, and carbonates. Various spectroscopic analyses [39,40,41,42] have revealed that the main compounds (>10 wt.%) of BA are SiO_2_, CaO, Fe_2_O_3_, and Al_2_O_3_, whereas Na_2_O, K_2_O, MgO, and TiO_2_ are present in minor concentrations (0.4–5.0 wt.%); thus, oxides are predominant. SiO_2_ is a predominant compound in BA, accounting for up to 49% [24].

**Figure 2:**
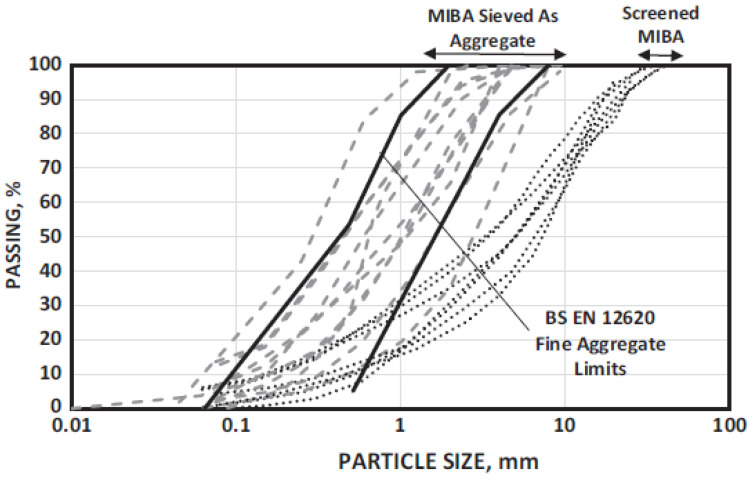
Particle-size distribution of MSWI BA [27].

Compared with natural sands and aggregates, MSWI BA is a relatively lightweight material. Figure 2 shows the range of the particle size for MSWI BA in comparison with that for fine aggregates of concrete (BS EN 12620:2013). In agreement with the morphological properties, a high water-absorption capacity has been reported for municipal incinerated BA (MIBA)—ranging from 2.4% to 15.0%, with an average value of 9.7% [27]. Siddique (2010) reported that combustor ash is highly absorptive, with absorption values ranging from 5% to 17% for fine particles and from approximately 4% to 10% for coarse particles. The bulk specific gravity values range from 1.5 to 2.2 for fine aggregate particles (0.075–4.75 mm) and from 1.9 to 2.4 for coarse aggregate particles (>4.75 mm), compared with approximately 2.6 to 2.8 for conventional aggregate materials [25]. Lynn et al. (2016) reported an average specific gravity of 2.32 for 35 samples, with a range of 1.8 to 2.8. Additionally, they reported that the loss on ignition (LOI), i.e., the weight loss of the material due to a temperature rise, was 5.8%, with a coefficient of variation of 71% [27]. A study in Denmark [2] indicated that the mean value of the BA LOI varies from 1.9% to 6.3%, depending on the efficiency of the incineration process.

The most abundant elements in MSWI BA are Ca, Si, Fe, and Al, and toxic elements such as Zn, Cu, Pb, Cr, Ni, Cd, and As are present in smaller quantities [27]. The BA has a pH ranging from 10.5 to 12.2, partly owing to the formation of hydroxides from CaO [2]. The presence of metallic Al is one of the most significant obstacles to BA utilization in Portland cement concrete (PCC), owing to the evolution of H_2_ gas originating from the reaction of metallic Al [2,8,25,43,44,45,46]. However, if the BA is separated from grate-shifting ash having a higher metallic-Al content, this problem can be mitigated [2].

**Figure 3:**
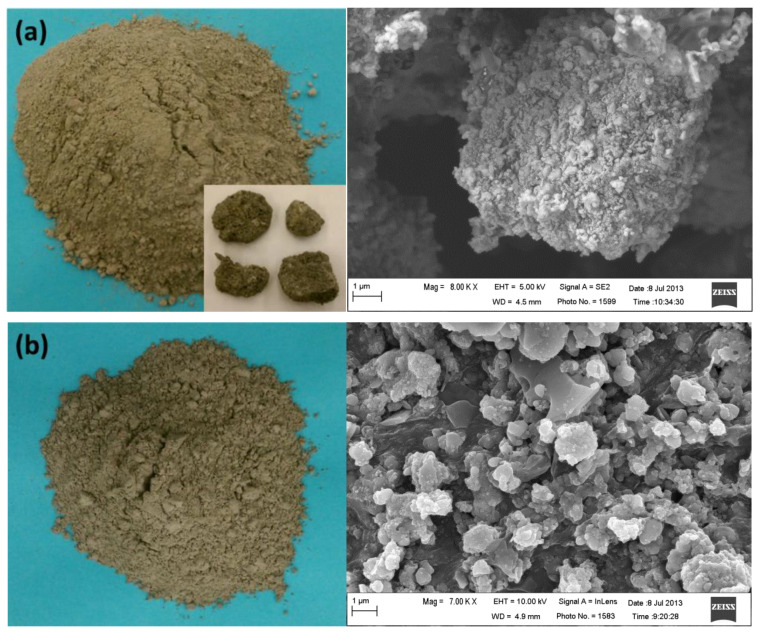
MSWI ashes: (**a**) BA; (**b**) FA.

Although BA (Figure 3a) contains a large amount of heavy metals, owing to its relatively low leaching potential, it is often considered as a benign material. The aging and weathering processes of BA can further reduce the reactivity and potential for heavy-metal release via the reaction between CO_2_ and water, which forms stable complex compounds in BA [47,48,49,50,51]. Additionally, aging can transform metallic Al into stable Al_2_O_3_, reducing the potential for H_2_-gas formation [2,52]. Therefore, the aging and weathering of BA can eventually improve its quality, making it viable as a construction material.

#### 3.1.2. MSWI FA

MSWI FA constitutes approximately 3% of the byproducts of MSWI and is characterized by fine particulate matter and a dusty appearance, with a gray or dark gray color [2,53]. Figure 3b shows a sample of MSWI FA, along with an SEM image. The physical properties of MSWI FA have been reported. The density of MSWI FA is lower than those of other fill materials used in the construction of embankments: typical values for MSW FA are 1.7 to 2.4 kg/m^3^, and that for sand is 2.65 kg/m^3^ [54,55]. Additionally, the LOI of MSWI FA at 975 °C is high (13%) [56].

MSWI FA is generally considered to be more toxic than MSWI BA, because FA has higher concentrations of heavy metals, salts, and organic micropollutants owing to the volatilization and condensation of different elements during the incineration [24,57,58]. Many studies have been performed on the chemical components of MSWI FA [37,59,60,61,62]. Table 1 presents typical chemical compositions of MSWI FA reported in the literature [62]. The main oxide components of the FA are SiO_2_, CaO, and Al_2_O_3_, in decreasing order of the amount. The FA also contains large amounts of Cl, Na, and K, and the most abundant heavy metals are Zn and Pb. Owing to the presence of highly soluble salts, Cl, and heavy metals, the FA is not considered for direct utilization as a construction material [2,3,5,24]. In particular, the high Cl content of the FA may increase the corrosion probability of reinforced concrete structures when the FA is mixed with cement. Additionally, when FA with lime scrubber treatment is incorporated into construction materials, the workability is significantly reduced owing to the high water-absorption capacity of hygroscopic CaCl_2_ [2]. Moreover, similar to the BA, the large content of metallic Al in the FA limits the applicability of the FA [63,64,65].

To reduce the adverse effects of FA, various treatment techniques are used: (1) extraction and separation using water or acids [5]; (2) chemical stabilization using carbon dioxide/phosphoric acid (CO_2_/H_3_PO_4_), ferrous sulfate (FeSO_4_) [5], sodium sulfide (Na_2_S) [5], and orthophosphate (PO_4_^3−^) [2]; (3) solidification using lime, cement, asphalt, and gypsum [5,8]; and (4) thermal treatment, e.g., vitrification or pyrolysis [2,5].

### 3.2. Hot Mix Asphalt (HMA) Applications

#### 3.2.1. Designs and Properties

MSWI BA and FA have been investigated for use in HMA mixtures. In a 2019 experimental study, the addition of MSWI FA reduced the penetration, phase angle (δ), and creep rate (m) and increased the softening point, complex shear modulus (G*), rutting factor (G*/sinδ), and creep stiffness (S) of the bitumen, suggesting that MSWI FA has significant potential for improving the performance of the asphalt binder [66]. Additionally, various fundamental physical tests have been performed to investigate the reuse of BA in pavement, and in a recent study, up to 80% of the natural fine aggregates could be replaced with MSWI BA, indicating that the asphalt mixture had good engineering properties, such as stability and indirect tensile strength [67,68]. Subsequently, implementation studies were performed to establish the relationship between the use of MSWI BA and the corresponding design methods for field applications [69,70,71,72]. Several studies indicated that MSWI BA has considerable potential for use in asphalt road pavement systems. However, MSWI BA tends to be used to replace fine aggregates rather than coarse aggregates. MSWI BA can reliably improve the resilient modulus, tensile strength, and fracture behaviors of the asphalt mixture when it is added as a filler material [29,73,74,75,76]. A study was performed in Florida to evaluate the mechanical performance of an HMA mixture containing MSWI BA, including the stability, flow, and indirect tensile strength. Furthermore, the optimum binder content and the optimum replacement ratio of MSWI BA were investigated via the Marshall mix design method. In that study, 20% replacement of the fine aggregate with MSWI BA yielded the optimal performance of the HMA [77]. Replacement of up to 20% yielded good resisting performance of the HMA with regard to compression, rutting, and softness; however, the binder content was increased with the MSWI BA content to satisfy the void limitation of the Marshall test, which was 3% to 5% [77,78]. Another study involving the rutting resistance performance indicated that MSWI BAs produce a relatively low rutting resistance; thus, the replacement of up to 10% of the surface mixture and up to 20% of the base course with BA are recommended [79,80]. Other studies [81,82] focused on the dynamic creep, moisture susceptibility, and low-temperature binding performance, revealing that MSWI BA improved the resistance of permanent deformation and provided satisfactory performance with replacement of up to 30% of the aggregate [81,82]. The MSWI BA content significantly affected the creep and viscous behaviors of the HMA mixtures [83].

The tensile strength of the HMA mixture is affected by the elastic modulus of the interfacial zone between the MSWI BA and the binder, which depends on the aggregate type of the BA [84]. Therefore, the tensile strength can be adjusted by selecting the type of BA and the substitution ratio. It was reported that 60% MSWI BA yielded the largest increase in the tensile strength without causing brittleness or fracture problems [85]. In another experiment, MSWI BA improved the tensile strength of the mixture, with adequate stability, a high flow rate, and a low void in mineral aggregate value [86]. Another study indicated that BA improves the adhesive force between the asphalt binder and the aggregates, enhancing the stability and indirect tensile strength [68]. An in-depth study on the bonding and damage mechanisms between the BA and the binder material was performed using the finite-element method [74]. Additionally, the stress–strain behavior of asphalt-stabilized MSWI BA was investigated [87]. Regarding the moisture susceptibility and raveling potential, the optimum content of MSWI BA was determined to be up to 15% for a surface course mixture and up to 20% for a base course mixture [88]. Romeo et al. (2018) reported that the MSWI FA can also significantly affect the tensile strength as a filler in the HMA mixture, with values ranging from 91% to 115% relative to that of a traditional filler [73]. Beyond the traditional method, i.e., the Marshall design procedure, the effect of MSWI BA on the properties and design of HMA based on the SUPERPAVE mix design procedure was studied, including the optimum binder content and aggregate gradation [78].

One of the critical factors limiting the use of MSWI ashes is their heavy-metal content. However, several studies indicated that the asphalt binder plays a role in solidifying and stabilizing the heavy-metal components in the MSWI ash in diverse mixture designs, such as HMA and stone matrix asphalt based on the SUPERPAVE and Marshall mix design procedures [89,90,91,92]. As such, MSWI BA and FA can partially replace fine aggregates in the HMA mixture in accordance with the chemical compositions of the ashes and after appropriate treatments, and acceptable performance and engineering properties for use in asphalt pavement systems are expected.

#### 3.2.2. Field Applications

The field applications of MSWI ashes and their performance have been investigated. The major facility for the field application of the ashes is the asphalt pavement system; however, there have been applications to other facilities, including parking lots, banks in canal structures, and runways of airports [2,93,94]. MSWI BA and FA have been used in both base and surface courses in HMA field applications. In a 2017 paper, Sormunen and Kolisoja recommended the use of MSWI BA as a lower structural layer of the asphalt pavement rather than a wearing layer for enhancing the durability of the road system [95].

In several field studies, the use of MSWI BA as an alternative aggregate for HMA mixtures yielded reliable performance for short- (2 to 3 years) to long-term (up to 20 years) monitoring periods [3,96,97,98,99]. In these cases, the bitumen effectively encapsulated not only the BA [97,100] but also the FA [73,91]. Regarding the replacement portion of the coarse aggregate, 50% to 100% replacement yielded excellent performance in multiple case studies [3]. In a study performed in Tampa, Florida involving the replacement of the sand component, up to 10% replacement of the fine aggregate yielded the same performance as a standard mixture, but replacement of >10% degraded the field performance [3]. Although the use of MSWI BA as an alternative to coarse and fine aggregates in HMA has generally yielded acceptable performance, in a few cases, it had poor performance and was repaved [3].

Furthermore, MSWI BA was used for the wearing layer (i.e., surface course) of the asphalt pavement system [3,79,101,102,103]. Application of MSWI BA to the surface course yielded excellent performance, with a high potential for large friction [102]. In most cases, although there were no difficulties in construction, the amount of initial voids in the mixture was larger than normal [67]. However, this difference in the voids was hardly examined with regard to the construction quality and engineering performance. Up to 20% substitution with MSWI BA yielded a mixture with not only a well-developed aggregate structure that could resist compression, rutting, and softness but also enhanced mechanical properties [77,78].

Although the U.S. and several European countries have empirically led the successful practice of exploiting MSWI ashes as unbound materials in road construction, direct mixing of the MSWI ashes in the bituminous mixture and field applications and performance monitoring works have rarely been pursued [104,105,106]. In a study conducted in France, MSWI BA was used as an unbound granular material for subbase construction and exhibited sound performance for 20 years, and the site exhibited a California Bearing Ratio (CBR) value of >120%, according to a falling-weight deflectometer (FWD) measurement [107]. In the U.S., MSWI BA has been used to replace gravel as the subbase material; it was used alone or mixed with natural gravel for parking lots and road pavement systems, and the constructed facilities exhibited good physical condition [3].

Although several studies have addressed the potential of and methods for replacing the natural aggregates in the asphalt mixture with MSWI BA and FA [38,108,109,110,111,112], fewer field implementations and long-term performance studies have been reported compared with the past century. Table 2 presents a summary of the field applications and performance monitoring for cases of direct mixing of MSWI ashes with bituminous binders through the replacement of the natural aggregates for flexible pavements constructed in the U.S. over the past century. Although the performance of road pavement systems was evaluated via visual investigation rather than via mechanical testing methods such as FWD measurement, the overall performance levels were acceptable. Unfortunately, however, few cases of short- and long-term performance monitoring have been reported in recent years.

### 3.3. Cement and Concrete Applications

#### 3.3.1. Cement Production (Cement Clinker)

MSWI ashes have been investigated as substitutes for raw (or feeding) materials for cement clinkers, replacing up to 100% of the raw material before sintering, which yields an ash–clinker material [40,113,114,115,116,117,118,119,120,121,122,123]. One of the primary reasons to use MSWI ashes in the cement-clinker production process is that the chemical compositions of MSWI ashes are similar to those of the raw cement clinker materials, including lime (CaO), silica (SiO_2_), alumina (Al_2_O_3_), iron oxide/hematite (Fe_2_O_3_), and calcium sulfate (CaSO_4_) [124,125]. According to previous studies, there are several ways to utilize MSWI ashes for cement-clinker production. The first approach involves replacing the raw material of the cement clinker with MSWI ash “as is” [113,114,115]. Although the MSWI ash may be subjected to sieving and drying, its chemical composition is not changed. As described in the previous section, the physical and chemical properties of MSWI ashes vary depending on the MSWI process. The use of RDF-based MSWI ashes has yielded better properties than that of MB-based ashes [113,114,115]. The MB-based ashes generally contain more metallic and nonmetallic elements that can degrade the properties of the cement clinkers [113,114,115,120,121,122,123]. The second approach involves washing the MSWI ash before using it as a replacement for some of the raw materials in the cement-clinker production process [116,120,121,122,123]. The prewashing of the MSWI ash can reduce the contents of detrimental components such as Cl, alkalis, sulfate, and heavy metals, [116,122]. The third approach involves applying either chemical or thermal treatment to MSWI ash for removing potentially detrimental elements. The effects of washing the ashes with acid or alkali water and mixing the ashes with a chemical agent (e.g., CaCl_2_) on the cement-clinker production have been investigated [40,117,119,123]. Lastly, additives have been combined with MSWI ash and raw materials of the cement clinker as correctives of SiO_2_, Al_2_O_3_, CaO, etc. [118,119,120]. Kikuchi (2001) added sewage powder, Al dross, and Cu slag to MSWI ash in cement-clinker production [115]. Ghouleh et al. (2018) employed waste lime, hydrated lime, and silica sand as additives for an MSWI ash–cement clinker [118].

Table 3 presents a summary of studies in which four different approaches were employed for utilizing MSWI ash in cement-clinker production. The MSWI ashes were collected from different locations and at different times [40,113,115,116,117,118,119]. The standard composition, i.e., the requirement of raw materials for Portland cement (ASTM C150-18) [126], is presented for comparison with the ash–cement clinkers. The cement clinker made with RDF ash had a higher compressive strength at 1, 7, and 28 curing days than the cement clinker containing MB ash [113,115], indicating that the cement clinker with RDF ash had larger amounts of cementitious or pozzolanic components (or both). Similarly, the cement clinker made with washed MB FA had a higher compressive strength than the cement clinkers made with unwashed MB and RDF. As indicated by Table 3, the ash–cement clinkers partially satisfied the requirements for Portland cement, and all the ash–cement clinkers had high contents of calcium and silicon oxides, which are primary components of Portland cement. As such, quality control with uniformity is currently a challenging issue for the use of MSWI ashes as additives in cement production owing to the different chemical compositions of MSWI ashes from diverse sources (i.e., MSWI plants). However, the foregoing studies on the chemical compositions of the ashes indicate that the ashes have considerable potential as additives in the production of cement. Therefore, if quality-control guidelines with in-depth ingredient analysis for MSWI ashes are established and the mixing ratio of the supplied ashes can be properly portioned and blended, MSWI ashes can be widely utilized in the construction industry.

#### 3.3.2. Cement Paste (Blended Cement)

**Figure 4:**
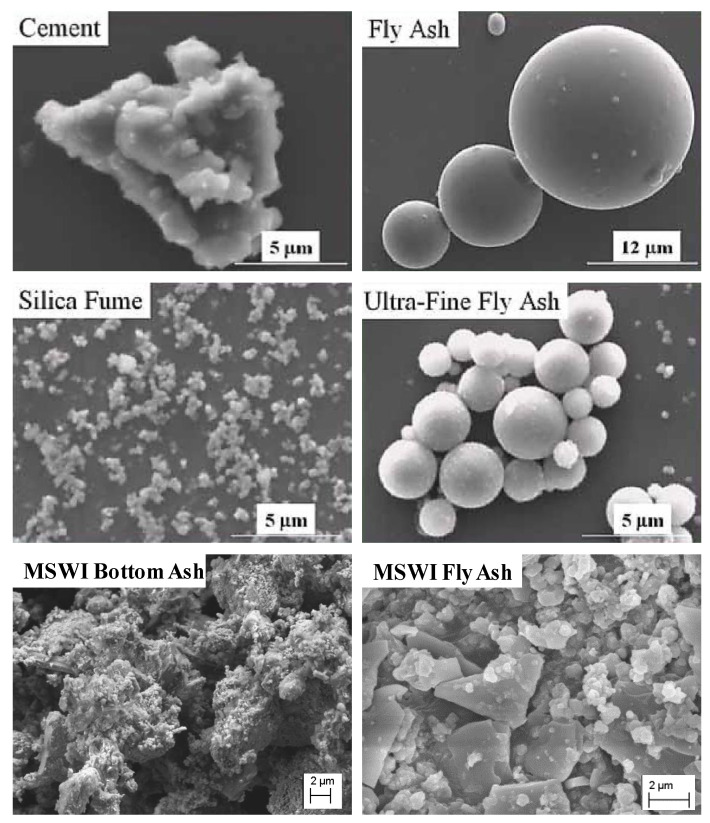
SEM images of MSWI BA, MSWI FA, and SCMs [130].

Blended cement comprising Portland cement and supplementary cementitious materials (SCMs) is common in the construction sector [124,125]. By replacing (fully or partially) Portland cement with SCMs, the energy, cost, and carbon-dioxide emissions (the main cause of global warming) can be reduced [124,125]. Additionally, appropriately designed blended cement can provide short- or long-term strength and improved workability [127]. Therefore, many researchers have attempted to incorporate MSWI ashes into Portland cement as SCMs [62,128,129,130,131,132,133,134,135,136]. MSWI ashes generally have high contents of calcium oxide and silicon dioxide, which are the main chemical components of Portland cement [120,121,122,123]. An et al. (2017) compared the microstructures of SCMs, MSWI BA, and MSWI FA [130]. Figure 4 presents a comparison of SCMs, BA (after grinding), and FA. The MSWI BA exhibits high porosity, as well as crystallinity and angularity, with vesicles connected to the exterior of the particles. The MSWI FA exhibits planar, elongated, and angular shapes, as well as clusters of sintered particles.

Several methods have been reported for producing ash-blended cement [62,128,129,130,131,132,133,134,135]. The first approach involves mixing Portland cement with MSWI ash “as is” [62,128,130]. The second approach involves applying prewashing or magnetic separation to the MSWI ash before it is mixed with Portland cement. This approach can remove chloride, alkalis, and metallic components that degrade the performance of the cement [129,135]. The third approach involves the enhancement of the MSWI ash through mechanical, chemical, or thermal treatment before the MSWI ash is combined with Portland cement [132,133].

Table 4 presents the chemical compositions of MSWI ashes that were used for ash-blended cement and Portland cement. Some of the MSWI ashes were mixed with Portland cement “as is,” and others MSWI ashes were combined with Portland cement after preprocessing, e.g., prewashing, magnetic separation, or chemical treatment. Additionally, the ASTM standard requirements are presented, for comparison. The ASTM standard for coal FA and raw or calcined natural pozzolan for use in concrete (ASTM C618) and the ASTM standard for blended cement (ASTM C595) have several chemical requirements for pozzolanic materials as well as blended cement [137,138]. As indicated by Table 4, some MSWI ashes have a high sulfur trioxide content and LOI. A high sulfur trioxide content in cement can cause expansion of the specimen [134], reducing the compressive strength. A high LOI of MSWI ash may support the existence of pre-hydration and carbonation, reducing the cementation or the pozzolanic reaction [124]. Table 4 also presents the water demand of ash-blended cement. The water demand of ash-blended cement is generally higher than of typical Portland cement, because MSWI ash is highly porous [113,114,115]. The setting times of ash-blended cements are longer than those of Portland cement, as shown in Table 4. These results indicate that the presence of MSWI ash in the ash-blended cement slows the hydration process owing to the reduced amount of PC in the mixture [62,128,129,130,131]. Although some MSWI ashes have high CaO and SiO_2_ contents, the crystal structures of those oxides may differ from that of Portland cement; thus, pastes made of ash-blended cement likely provide lower compressive strength than ordinary Portland cement paste (see Table 4). Regarding the strength, ash-blended cements containing RDF BA or MB BA that have undergone a magnetic separation process have a higher compressive strength than ash-blended cements containing MB BA “as is.” This indicates that MSWI BA with low contents of harmful elements (e.g., alkalis and metallic components) can be favorable for strength development. However, in the case of MSWI FA, the difference in compressive strength between RDF FA and MB FA is insignificant. For MSWI FA, the amount of FA in the ash-blended cement is more important than the pre- or post-processing treatment. This phenomenon is explained by the “filling effect.” An optimal amount of fine dust can facilitate the strength development of cement composites by filling the pores [125,130].

#### 3.3.3. PCC

MSWI ashes have been utilized in concrete as replacements for either Portland cement or aggregates. There are many examples of SCMs and recycled aggregates being adopted in the concrete industry [124,125,127] as replacements for Portland cement and natural aggregates. Coal FA, silica fume, ground granulated blast-furnace slag, and metakaolin are well-known SCMs [127]. Coal FA is a great example. The American Coal Ash Association reported that approximately 38.2 million tons of coal FA is produced annually in the U.S., and half of the total coal FA (approximately 19.2 million tons) is reused in cement/concrete applications [139]. Similarly, MSWI ash has potential as an SCM and as a substitute for aggregates in concrete.

Extensive efforts have been made to reuse MSWI FA and BA in concrete as replacements for either Portland cement or fine and coarse aggregates [27,130,140,141,142,143,144,145,146,147,148,149,150]. Because fine and coarse aggregates occupy 70–80% of the total volume in concrete, it can be advantageous to replace some of the aggregates with MSWI ash [125]. Table 5 presents examples of studies on the use of MSWI ash in concrete. As mentioned in the previous section, the water absorption of MSWI ash is high; thus, the water/binder ratio must be relatively high for maintaining sufficient workability of the ash-mixed concrete. Nevertheless, most ash-mixed concretes exhibit low slump values in comparison with the control mixture. In the mix design of ash-mixed concrete, a relatively low replacement ratio of up to 50% was used for the replacement of Portland cement, while the ratio reached 100% for the replacement of aggregates. Most ash-mixed concretes had a lower compressive strength than the control mix. However, in a few cases, the ash-mixed concretes had comparable (or slightly higher) compressive strengths. In a few studies, MSWI ash had a beneficial on the concrete. MSWI ash has a high silicon-dioxide content and a good ratio of silicon dioxide to calcium oxide [130,144,149]. In general, the ratio of silicon dioxide to calcium oxide for Portland cement is 1:3 [124].

In summary, extensive studies on the use of MSWI ashes in hydraulic cementitious composites have been performed, and the results indicate that the ashes have considerable potential for application to civil infrastructures such as road base systems. Nonetheless, few studies involving field applications or short- and long-term performance monitoring have been performed [28].

Other observations have been reported, particularly regarding side effects in concrete. It has been reported that metallic elements—particularly metallic Al—in MSWI ash can generate H_2_ gas in concrete [31,130,143,146]. Under high-pH conditions, metallic elements can be partially corroded. Consequently, some alkali metals (e.g., Al) can evolve H_2_ gas in concrete. The gas generated in the concrete can cause volume expansion of the concrete and increase the porosity of the concrete. As indicated by Table 5, some of the ash-mixed concretes underwent volume expansion [130,143], and their increased porosity values were reported [130,143,146]. Ultimately, the gas evolution affects the reduction in the strength of ash-mixed concrete. Thus, researchers have proposed prewashing MSWI ash with either acid or alkali water [117,150]. Through the prewashing process, metallic elements in the MSWI ash can be stabilized or eliminated before the ash is used in the concrete matrix.

### 3.4. Geomaterial Applications

As candidate road base, subbase, and/or subgrade materials, MSWI BA and FA were examined to determine their physical (moisture content, apparent and bulk densities, crystallinity, etc.) and engineering properties (particle-size distribution, abrasion and impact resistance, etc.). It was concluded that MSWI BA is more adaptable than MSWI FA for road construction [11]. The shear strength, elastic modulus, and bearing capacity of MSWI BA were reported to be similar to those of natural sand [28]. Another study suggested that MSWI ash can significantly improve the engineering properties of sand, including the unconfined compressive strength and shear strength [151]. MSWI BA has been used in the field. Huang et al. (2020) applied MSWI BA as an alternative to subgrade materials for HMA pavements, and the results indicated that the tensile strain at the bottom of the HMA layer was diminished, along with the compressive strain on top of the subgrade layer [152]. MSWI BA was used as an unbound granular subbase in France; the road pavement has been soundly in use for 20 years, and it exhibited a CBR value of >120%, according to FWD measurements [107]. MSWI BA was also used as an aggregate substitute in an asphalt-stabilized base course in New Hampshire, U.S., and a two-year study revealed that the relatively constant hot-mix formulations satisfied the specifications and that the bitumen effectively encapsulated the MSWI BA [6]. In Houston, Texas, MSWI BA was used as a base course with asphalt stabilization and then surfaced by conventional HMA, and according to three years of monitoring, the test section had excellent performance, with only minor cracks on the surface layer [8]. Additionally, it was demonstrated that MSWI BA is an adequate alternative to soil for embankments and landfills and a suitable material for granular layers (bases and subbases) [106,153]. Patil et al. (2016) reported that replacement with 15% MSWI ash may enhance the strength of the natural soil subgrade in the road construction sector [154].

It was also reported that the addition of 25% MSWI BA stabilized the geotechnical properties of a clayey soil, including the unconfined compressive strength and CBR [155]. For application as a filling material in landfills, MSWI BA can be used with cement stabilization. Singh and Kumar (2017) investigated the applicability of cement stabilized with MSWI ashes as a lightweight filling material in different infrastructures, such as embankments and road subgrades [156]. Other examples of field applications of MSWI BA in European countries and the U.S. are presented in Table 6. Many studies have demonstrated the potential applications of MSWI ashes, with a focus on the geotechnical behavior. The results indicate that MSWI ashes are applicable in various fields of civil construction, e.g., roads, embankments, concrete, and geotechnical applications [157]. Despite their potential, MSWI ashes have seldom been applied as geomaterials in the field, and performance monitoring is rarely conducted for construction projects. Although a multi-criteria decision-making method to select the optimal MSWI materials for obtaining the ideal embankment material was reported in 2020 [158], it is challenging to determine the suitability of MSWI materials case-by-case, owing to the diverse compositions of MSWI ashes from different sources.

**Table 4:** Comparison of the chemical compositions and physical and mechanical properties between ash-blended cement and Portland cement.

Property	RDF FA (J. D. Hamernik et al. 1991) [128]	MB FA (S. Remond et al. 2002) [62]	MB FA (Z. Yang et al. 2018) [135]	MB BA (X. G. Li et al. 2012) [129]	RDF BA (J. An et al. 2017) [130]	MB BA (Z. Yang et al. 2018) [135]	Portland Cement [137,159]	Chemical Requirements for Pozzolanic Material
Treatment	N/A	N/A	Prewashing	Magnetic separation	N/A	Magnetic separation	N/A	
SiO_2_, %	38.03	27.23	4.5	59.59	15.8	53.8	21.49	SiO_2_ + Fe_2_O_2_ + Al_2_O_3_, (Class N pozzolan: 70 min., Class F: 50 min., Class C: 50 min.) CaO, (Class N pozzolan: report only, Class F: 18.0 max., Class C: 18 min.) SO_3_, (Class N pozzolan: 4.0 max., Class F: 5.0 max., Class C: 5 max.)
Al_2_O_3_, %	14.65	11.72	1.6	18.61	5.7	8.7	4.21	
Fe_2_O_3_, %	3.43	1.80	-	5.50	3.61	-	3.50	
CaO, %	20.17	16.42	60.6	7.58	48.6	14.3	64.90	
MgO, %	2.19	2.52	1.7	1.32	3.06	1.9	-	
SO_3,_ %	2.82	3.00	5.5	0.65	9.96	0.3	0.70	
Na_2_O, %	2.47	5.86	0.3	1.32	5.63	11.7	-	
K_2_O, %	0.74	5.80	0.5	2.29	1.13	1.1	-	
ZnO, %	-	-	-	-	0.75	-	-	
Cl, %	-	7.2	1.3	-	2.2	0.6	-	
Other, %	5.4	1.46	0.6	-	6.61	1.7	5.2	
Loss of ignition, %	10.10	13.0	20.3	0.43	-	-	-	LOI, (Class N pozzolan: 10 max., Class F, C: 6 max.)
SiO_2_ + Fe_2_O_2_ + Al_2_O_3_	56.11	40.75	6.1	83.7	25.11	62.5	29.2	
Percentage of cement replacement (%)	35% replacement	5–20% replacement	10–50% replacement	10–50% replacement	10–100% replacement	10–50% replacement		
Water/Cement	Depends on water demand	0.5	Depends on water demand	Depends on water demand	0.5	Depends on water demand	0.5	
Water demand (%)	30.27	-	29.5–35.8	26.0–29.3	-	27.3–23.3	26.2–32.0	
Setting time, mins (initial and final)	Initial: exceed 1728	Initial: 420–1860, Final: 540–2460	Initial: 235–210, Final: 293–282	Initial: 206–258, Final: 258–350	Initial: 313–240, Final: 546–1860	Initial: 207–246, Final: 269–302	Initial: 26.7–264, Final: N/A	
28-d compressive strength, MPa, (%, ratio to control)	26.95 (97.3%)	62 (107%)–55 (95%)	46.5 (73.8%)–17.1 (27.1%)	45.9 (88%)–34.6 (66%)	15.4 (76%)–2.8 (10%)	53.2 (84.4%)–29.1 (46.2%)	38.9–58.4	

**Table 5:** Chemical compositions and physical and mechanical properties of ash-blended concrete.

		MB BA (J. Pera et al. 1997) [141]	MB BA (N.B. Chang et al.1999) [142]	RDF BA (N.B. Chang et al.1999) [142]	MB FA (J. Aubert et al. 2004) [143]	MB BA (B. Juric et al. 2006) [144]	RDF BA (G. Wegen et al. 2013) [146]	RDF BA (A. Abba et al. 2014) [147]	RDF BA (J. An et al. 2017) [130]	Normal Concrete [159]
Type of ash		BA	BA	BA	FA	BA	BA	BA	BA	(Granite for coarse aggregate)
Type of replacement		Coarse aggregate	Fine aggregate	Fine aggregate	Cement	Cement	Coase aggregate	Coarse aggregate	Fine aggregate	-
SiO_2_ (wt.%)		54.6	18.6	19.8	20.67	24	-	42	15.80	-
CaO (wt.%)		11.1	34.6	44.6	25.23	39	-	18	48.60	-
Size		4–20 mm	0.075–4.75 mm	0.075–4.75 mm	6–200 µm	0.063–8 mm	2–12 mm	0–10 mm	0.075–4.75 mm	
Water absorption (%)		2.36	7.4	9	-	-	7.1–11	-	12.8	0.60
Fineness modulus		-	3.59	3.34	-	-	-	-	2.52	-
Density (kg/m^3^)		2.21	2.27	2.38	2.26	-	2.09–2.23	-	2.20	2.69
Specific area (m^2^/g)		-	-	-	2.26	-	-	-	-	-
Mix proportion (cement:fine:coarse)		1:3.21:3.17	1:2:4	1:2:4	1:2.73:4.15	1:3:0 (Mortar mix)	1:2:3.8	1:2.2:2.5	1:2.11:2.64	1:2.08:2.29
W/B (binder)		0.63	0.7	0.7	0.73	0.55	0.5	0.75	0.5	0.5
Level of replacement		50% and100% of coarse aggregate	100% of fine aggregate	100% of fine aggregate	12.5% and 50% of cement	5–40% of cement	20% of coarse aggregate	50% of fine gravel	10–50% of fine aggregate	-
Slump (mm)		145	10	20	65 and 75	55	150	similar to the control	130–200	150
Compressive strength, MPa, (%, ratio to control)	3 d	-	5.77 (52.6%)	8.95 (81.59%)	-	-	20.0 (90%)	-	-	-
	7 d	20.8 (81.4%)–17.0 (60.7%)	7.22 (43.83%)	10.54 (64.03%)	26 (81.3%)–6 (18.8%)	34.7 (109%)–26.3 (69%)	-	-	-	22.3
	28 d	29.9 (90.9%)–22.3 (67.8%)	10.70 (45.16%)	15.31 (64.65%)	33 (82.5%)–8 (20%)	49.5 (99%)–39.8 (79%)	39.5 (84%)	22.70 (101.8%)	48.5 (95.6%) –21.8 (43.0%)	32.1
	90 d	34.6 (82.8%)–28.4 (67.9%)	-	-	29 (76%)–8 (21%)	-	47.4 (89.7%)	-	-	-
Shrinkage, ratio to control		250 µm/m, 76%	-	-	-	-	Smaller than control	-	Volumetric expansion	-
Porosity (%)		-	-	-	14.5–25.0	15.9–17.2	-	-	11.6–18.43	-

**Table 6:** Field applications of MSWI BA as a geomaterial and performance evaluation.

Location	Project Description	Performance Notes
Skaelskor, Denmark [28]	Unbound subbase materials for heavy traffic road pavements in 1976	In 2001, exhibited good condition with low rutting
Le Mans, France [41]	Unbound subbase materials for urban road in 1978	In 2001, exhibited good deflection, compaction, and grading properties
Linkoeping, Sweden [160]	Unbound subbase materials for road pavement in 1987	In 2006, exhibited suitable performance with stable stiffness
Umea, Sweden [161]	Unbound subbase materials for asphalt road pavement in 2001	In 2006, exhibited suitable performance
Milan, Italy [162]	20% BA mixed with gravel for road foundation constructed in 2005	In 2014, exhibited usable performance
Newcastle, UK [163]	Sand replacement protection liner at Burnhills landfill in 2001	Stable performance and positive shear properties
Rochester, U.S. [28]	Unbound subbase materials for low traffic access road	Normal performance after 1.5 years
Shelton, U.S. [164]	Filling materials for landfill road	Good structural properties with small settlement

## 4. Environmental Effects of MSWI Ashes as Construction Materials

### 4.1. Leaching Properties and Regulations

Owing to the presence of heavy soluble salts and heavy metals in MSWI ashes, the leaching properties of BA, FA, and combined ash are considered as critical parameters for utilizing MSWI ashes without damaging the environment [51,165]. Different countries have implemented their own standard procedures for leaching tests and have set threshold limits for toxic elements to evaluate the leaching potential of heavy metals and soluble salts when MSWI ashes are either landfilled or in contact with soil and water [1,2,3,4,5]. Leaching-test results revealed that FA has significantly higher contents of soluble salts (i.e., Na, K, Ca, Cl) and toxic elements (i.e., Pb, Zn, Cr, Ni, Cu) than BA [166,167]. Oxyanions, such as Zn and Pb, are amphoteric and are characterized by a high leaching potential under both high- and low-pH conditions. The release of such amphoteric heavy metals from FA can be significantly increased owing to the high pH of FA originating from APC devices containing a lime solution [168]. Substantial Pb leaching has also been reported by researchers in Korea and Japan [169]. Danish researchers [170] evaluated BA and FA from 25 MSWI plants from 1998 to 2010 and reported that the FA is likely to exceed the leaching limit values for Cl, SO_4_, Cd, Cr, Hg, Mo, Pb, and Zn, whereas the BA is likely to exceed the limit values for Cl, SO_4_, Cu, Mo, Sb, and Se.

The environmental regulations of several countries for MSWI ashes were compared. As mentioned previously, different countries have implemented their own leaching-test procedures depending on their regulatory and environmental perspectives, yielding wide variations in the threshold values for particular chemical constituents involved in MSWI-ash utilization. Different countries’ leaching criteria are compared in Table 7. In the U.S., BA and FA are mixed together and disposed of in landfills as combined ash [16]. According to the Resource Conversation and Recovery Act, MSWI ashes must pass the Toxicity Characteristic Leaching Procedure (TCLP) (SW-846 EPA Method 1311) [171,172] to be considered as non-hazardous waste. FA often fails the TCLP; however, BA with smaller amounts of hazardous constituents generally passes. Hence, in the U.S., FA and BA are combined and disposed of together to avoid the high cost and negative stigma of special disposal techniques for hazardous waste. An alternative leaching-test method has been developed to better simulate the field performance. The Synthetic Precipitation Leaching Procedure (EPA Method 1312) [3] has been applied using sulfuric/nitric acid (40/60 by weight), and the EPA has been working with the Leaching Environmental Assessment Framework (LEAF) to characterize leachates and runoff from the field [56]. LEAF tests are designed to evaluate the dependence of the pH and mass transfer rate on a batch or column leaching tests with different liquid-to-solid ratios [56]. In the Netherlands, the regulatory framework Federal Waste Management Plan (Landelijk Afvalbeheer Plan, LAP) does not permit the mixing of BA and FA [3,4], and the Dutch Waste Incineration Directive requires the LOI to be <5% [4]. According to the standard column leaching test (NEN 7343, liquid/solid ratio (L/S) = 1–10) [173], two utilization categories are distinguished for the application of BA to a layer with a maximum depth of 15 m [4]. In Denmark, the European Committee for Standardization (CEN) sets three categories (1–3) based on the leaching criteria corresponding to the compliance standard batch leaching test (CEN prEN 12457, L/S = 2) [174]. Categories 1 and 2 have strict leaching criteria, and category 3 has lenient criteria. MSW ash is categorized as soil and inorganic residue; BA mostly falls under category 3 and never falls under category 1, owing to the large amounts of inorganic constituents. Germany has encouraged research and development in an effort to improve the treatment techniques, separation schemes, and beneficial utilization of MSWI ash [3,4]. According to the German regulation set by Board of German States of Ministers (LAGA) [4], the BA must satisfy the standard leaching criteria based on the batch leaching test (DEV S4, L/S = 10) [175], and the total organic carbon content should be <1.0 wt.%. In France, a standard batch leaching test (NF X31-210, L/S = 20) [176] was designed to classify the BA into three categories for utilization in civil-infrastructure projects. After nine months of maturation, the BA mostly fell under the first category, exhibiting the lowest leaching potential [4]. However, to utilize the BA with nine-month maturation, additional requirements must be satisfied [4].

### 4.2. Reduced Leachate for HMA and PCC Applications

Leaching tests have been conducted to evaluate the release of toxic elements from the leachate of BA, FA, and combined ash when the ash was used as a base or subbase course in asphalt pavement [105,106,180,181,182,183,184,185,186,187,188], PCC products [43,64,129,143,165], and embankment fills [189,190,191]. In U.S. studies, the heavy-metal concentration in the leachate mostly satisfied the leaching requirements [171] and often satisfied even the U.S. drinking-water standard [178]. Additionally, it was reported that the dioxin and furan (particularly in FA) do not pose any threat to the environment or health [16]. However, although the heavy-metal concentrations in ash leachate were mostly found to be below the threshold limits in the U.S., the salt concentration was reported to be significantly higher than the limit of the drinking-water standard [7,170].

Several researchers confirmed a significant reduction in the leaching potential of BA, FA, and combined ash when they were combined with cement and concrete [106,129,165,182]. A study in Spain involved the formulation of a granulated material with combined ash and cement to use as a secondary building material [182]. In this study, batch leaching tests were performed to evaluate the leaching behavior of BA, APC, and combined ash containing concrete. Concrete mixtures were prepared with 10% cement, 10% APC, and 80% BA by weight. The test results are presented in Table 8, along with threshold values established according to the utilization criteria [192] and the three categories of landfill criteria [193] set by the Spanish Government for MSWI BA utilization as a secondary building material. A significant reduction in leaching was observed for the combined ash mixed concrete formulation, and the heavy-metal concentrations were below the criteria for utilization. A significant reduction in the leaching of heavy metals via concrete binding has been confirmed by many other researchers [106,129,165,180]. In the concrete application, cement (as a binder) captures the heavy metals physically and chemically, and they are transformed into more stable and insoluble compounds, making it less vulnerable to contamination [194]. Tasneem et al. (2017) reported that heavy metals from MSWI BA were significantly reduced by the concrete application. Here, MSWI BA was used as partial replacement for the fine aggregate (up to 50%). The concentrations of all the investigated heavy metals (except for Al) were lower than the Secondary Maximum Contaminant Level (SMCL) criteria.

**Figure 5:**
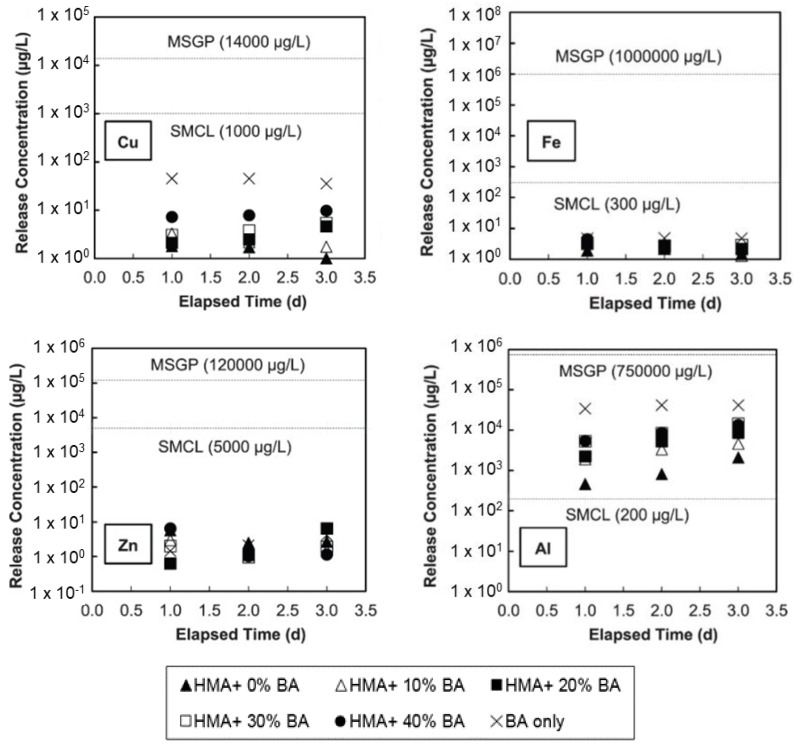
Leaching concentrations of priority elements, including Cu, Fe, Zn, and Al, from HMA containing BA compared with the SMCL and MSGP criteria [196].

The leaching potential of ash residue can be significantly reduced via physical encapsulation in asphalt [195]. Tasneem et al. (2017) investigated the leachate of HMA in contact with MSWI BA (as a fine-aggregate replacement) [196]. As shown in Figure 5, only the Al concentration exceeded the SMCL criterion; the concentrations of all the other heavy metals were lower than the SMCL criteria. Nonetheless, the Al concentration was lower than the Multi-Sector General Permit (MSGP) criterion Importantly, the MSWI BA used in the study had a high metallic-Al content, leading to significant H_2_-gas generation [150].

Over the past decades, cementation has been applied to MSWI FA. Li et al. (2001) reported that the use of cementitious material is the most widely employed technique for solidifying and stabilizing MSWI FA [197], because cement easily forms a durable and monolithic material and encapsulates hazardous components in MSWI FA under disposal conditions [198,199]. This cement immobilization is also the best method for disposing of hazardous and toxic waste, according to the American State Bureau of Environmental Protection [200]. Shi and Kan (2009) performed laboratory leachate tests on FA-mixed cement and concluded that cementation was effective for reducing the amount of leachate. Although MSWI FA causes damage, e.g., corrosion due to the high amount of Cl, the leaching toxicity is within a safe range [165].

## 5. Conclusions and Recommendations

We reviewed the current management practices for MSWI ashes and summarized their advantages as construction materials. The results indicated that MSWI ash can be employed in the construction sector. The comprehensive review focused on the following areas: (1) current management practices for MSWI ashes (in particular, a comparison between European countries and the U.S.); (2) the physical, chemical, and leaching properties of MSWI ashes; (3) the engineering properties and performance of ash-combined mixtures, particularly in HMA and PCC; and (4) environmental criteria and regulations. According to the extensive review of the characterization of MSWI ashes (both raw ash and ash-mixed composites) and performance evaluation and monitoring of their field applications, the following conclusions and recommendations are presented.

- Despite the large amount of MSWI ash generation and numerous positive research results related to the reusability of MSWI ash, the U.S. has a less active recycling program than European countries. In general, the U.S.’s current practice of MSWI management involves combining MSWI BA and FA, followed by disposal in landfills. Because most of the toxic substances come from the MSWI FA, the separate handling of the BA and FA would be more effective for the reuse process.
- The use of the ash in geomaterial applications, e.g., road subbase/subgrade and backfill materials, yields good engineering performance; however, leachates released by the direct contact with water (rainfall, runoff, surface water infiltration, etc.) pose environmental concerns.
- Reusing the MSWI ash in an HMA mixture can be a feasible option if the ashes replace <20% of the natural aggregate. Although few field applications have been reported in recent years, the field experience and short-/long-term performance over the past century in the U.S. indicate reasonable durability. However, this asphalt application requires the addition of an asphalt binder owing to the high absorption characteristics of MSWI ashes, which is not cost-effective.
- Incorporating the MSWI ashes into cement/concrete composites appears to be the most promising option because of the cementation effect, which results in not only a significant reduction in the release of toxic elements but also adequate structural integrity. MSWI ashes are also viable as raw materials for cement clinkers, SCMs, and aggregate replacement from an engineering-performance viewpoint.
- Considering the wide range of constituents of MSWI ashes depending on the source and feeding materials to incineration, proper chemical characterization of the ashes is necessary for the concrete application. Regarding the concrete’s filler materials, deteriorating substances such as metallic Al, Cl, and highly soluble salts can be identified and selectively removed prior to the use of the ash in concrete. Weathering and/or proper pretreatment processes are recommended for diminishing side effects such as H_2_-gas emission in the cement/concrete application.
- The development of standardized quality-control protocols, along with in-depth ingredient analysis and blending manuals for MSWI ashes from diverse sources, is recommended for promoting the application of MSWI ash in the field.

## Figures and Tables

**Table 1:** Example chemical composition of MSWI FA [62].

Compounds			Heavy Metals	
Compound	Mass Fraction (%)		Metal	Content (mg/kg)
LOI (975 °C) SiO_2_ CaO Al_2_O_3_ Na_2_O K_2_O MgO Fe_2_O_3_ TiO_2_ P_2_O_5_ Mn_2_O_3_ SrO Ba Cl SO_3_	13.00 27.23 16.42 11.72 5.86 5.80 2.52 1.80 0.84 0.34 0.05 0.01 0.22 7.20 3.00		Zn Pb Cu Mn Cr Cd Sn Sb Ni Se Te V Mo As Co Tl	11,000 4000 670 600 450 270 180 110 50 50 46 32 25 21 21 <5

**Table 2:** Field applications of direct mixing of MSWI ashes in the asphalt mixture and long-term performance evaluation of flexible pavement systems in the U.S. [3].

Location	Project Description	Performance Notes
Houston, Texas	300 feet (91 m) of demonstration pavement in 1974. 6-inch (15 cm) base course containing 100% ash aggregate, 9% binder, and 2% lime	The test section was reported to be in excellent condition in 1978 and again in 1993.
Philadelphia, Pennsylvania	Demonstration pavement in 1975. Ash replaced 50% of rock aggregate in a 90-foot (27 m) test section of 1.5 inch (3.8 cm) surface course. Binder content was 7.4%, and 2.5% lime was added.	The condition was reported to be acceptable in 1993.
Lawrence County, Kentucky	A one-mile bituminous surface with 40% MIBA as aggregates was placed in October 1987 on State Route 3 in Lawrence County, Kentucky	Exhibited excellent performance with a high potential for large friction [102]
Delaware County, Pennsylvania	A demonstration section was 60 feet (18 m) long and was placed in 1975 with 7% binder.	The condition was acceptable in 1993.
Washington, DC	Ash replaced 70% of the natural rock aggregate in one mixture and all the aggregate in a second. 400 feet (122 m) of 4.5 inch (11.5 cm) base course contained 9% binder and 2% lime.	The condition was good in 1993.
Lynn, Massachusetts	Ash paving demonstration on Route 129 consisted of five test sections placed during November 1980 over 1.5 miles (2.4 km) between Goodwin Circle and the beginning of St. Ann’s Cemetery.	The condition of the test section was good in 1993.
Tampa, Florida	McKaynite, a proprietary aggregate processed from ash, was used as an aggregate in asphalt paving in February 1987. 5%, 10%, and 15% of the sand component in three 500-foot (152 m) test sections was replaced with McKaynite. Up to 10% substitution yielded the same behavior as standard design mixes used for comparison. The monitoring was stopped after 1 year.	The road was still in place in April 1997 and was described as showing some wear but acceptable.

**Table 3:** Comparison of the chemical compositions and physical and mechanical properties between ash–cement clinker and Portland cement clinker.

Approach		MSWI Ash “as Is”		Prewashing Process	Chemical or Thermal Treatments		Additives	
Standard Composition Requirements	Portland cement (Type I & II)	RDF BA (E.J. Duckett et al. 1980) [113]	MB BA (R. Kikuchi. 2001) [115]	MB FA (L. Wang et al. 2010) [116]	MB BA (J. Pan et al. 2008) [117]	MB BA (X.C. Qiao et al. 2008) [40]	MB BA (R. Kikuchi. 2001) [115]	MB FA (Z. Ghouleh et al. 2018) [118]
Treatment or Additive		N/A	N/A	Prewash	Prewash with water and acid	Thermal treatment and calcium hydroxide	Sewage powder, aluminum dross, and copper slag	Waste lime, hydrated lime, and silica sand
Blend ratio (wt.%)	A	Replacing raw materials with ash up to 40% (optimum is 30%)	40.6:56.6:2.8 (ash:lime:clay)	79.48:15.88:2.58: 1.03:1.03 (ash:raw materials:SiO_2_:Al_2_O_3_:FeO_3_)	75.9:1.3:17.1:2.2:3.5 (lime:iron slag:clay:sand:ash)	90:10 (ash:Ca(OH)_2_)	27.5:54.0:6.9:10.4:0.9:0.3 (ash:lime:clay:sewage:aluminum:copper)	42.8:42.8:9.1:5.3 (ash:waste lime:hydrated lime:silica sand)
Chloride (wt.%)	F	0.01	0.4	0.0047	0.00037	-	0.3	4.23
Sulfate (wt.%)	3.0 (max. for both)	0.03	1.3	0.4	0.02	2.34	1.2	5.14
Calcium oxide, %	A	-	60.8	66.33	65.40	20.20	61.5	47.59
Silicon dioxide, %	20.0 (max. for II)	-	18.1	22.44	23.03	36.20	19.0	19.78
Aluminum oxide, %	6.0 (max. for II)	-	10.2	4.98	5.37	8.48	10.0	5.33
Ferrite oxide, %	6.0 (max. for II)	-	3.5	3.09	3.87	6.21	3.0	1.51
Magnesium oxide, %	6.0 (max. for both)	0.64	1.8	1.34	2.45	1.58	2.0	1.83
Loss of ignition, %	3.5 (max. for both)	-	-	0.24	-	12.80	-	3.26
Initial & final set time (mins)	Not less than 45 for initial	72, 198	40, 60	73, 125	260, 380	43, 62	30, 40	-
W/C ratio	A		0.5	0.5	-	0.5	0.5	-
UCS, MPa (1, 7, and 28 d) ASTM C109	10, 17 (min. for 3, 7 d)	9.5, 29.7, 46.2	5, 17, 40	13.4, 26.97, 55.43 (1,3, 28 d)	16.7, 26.5, 42.2	N/A, 12.7, 14.7	15, 22, 35	5.2, 15.8, 27.0
Fineness (cm^2^/g)	2600 min	-	-	-	3550	-	-	3630 ± 330

A: Not applicable; F: Limit not specified by purchaser.

**Table 7:** Leaching criteria for different constituents from MSWI BA residue for utilization in various countries (mg/L) [4,177,178].

Element	The Netherlands Column (L/S = 1–10) 2005	Denmark Batch (L/S = 2) 2000	Germany Batch (L/S = 5) 1994	France Batch (L/S = 5) 1994	US EPA Toxicity Criteria 1987	US Drinking Water Standard 2009
Cl	440	300	125	-	-	-
F	14.4	-	-	-	-	4
SO_4_	3250	400	300	500	-	-
Na	-	150	-	-	-	160
As	0.35	0.005	-	0.1	5	0.01
Ba	7.75	0.4	-	-	100	2
Pb	0.41	0.01	0.025	0.5	5	0.015
Cd	0.00305	0.004	0.0025	0.05	1	0.005
Cr	0.06	0.05	0.1	0.05	5	0.1
Cu	0.165/1.15 ^a^	0.2	0.15	-	-	1 ^b^
Hg	0.00375	0.0001	0.00005	0.01	0.2	0.002
Mn	-	0.1	-	-	-	-
Ni	0.175	0.007	0.02	-	-	0.1
Zn	0.7	0.15	0.15	-	-	5 ^b^
Co	0.115	-	-	-	-	-
Mo	0.13/1.15 ^a^	-	-	-	-	-
Sb	0.06/0.1 ^a^	-	-	-	-	0.006
Se	0.0135	-	-	-	1	0.05
Sn	0.115	-	-	-	-	-
V	4.8	-	-	-	-	-
Ag	-	-	-	-	5	0.1 ^b^
Tl	-	-	-	-	-	0.001

^a^ In The Netherlands, BA is considered to fall under a special category with relatively lenient leaching criteria for Cu, Mo, and Sb [4]. ^b^ Secondary drinking water standards [179].

**Table 8:** Leaching results for MSWI BA, APC residue, and the formulated concrete mixture (mg/kg) [182].

Element	BA	APC	Combined Ash	Concrete with Combined Ash	Criteria for Utilization ^b^	Criteria for Landfill ^c^		
						Inert	Non-Hazardous	Hazardous
As	0.003	0.004	0.003	0.001	1.0	0.50	2	25
Ba	0.504	43.682	5.302	15.04	-	20.0	100	300
Cd	0.043	0.040	0.043	0.026	1.0	0.04	1	5
Cr	0.390	3.643	0.751	0.050	5.0	0.50	10	70
Cu	0.989	4.999	1.435	0.938	20	2.00	50	100
Hg	<0.01	<0.01	0.010	<0.010	0.2	0.01	0.2	2
Mo	0.401	2.611	0.647	0.117	-	0.50	10	30
Ni	0.060	1.290	0.197	0.170	5.0	0.40	10	40
Pb	0.079	138.284	15.435	2.139	5.0	0.50	10	50
Sb	0.460	0.040	0.413	0.079	-	0.06	0.7	5
Se	0.007	0.092	0.016	<LOD ^a^	-	0.10	0.5	7
Zn	0.818	35.083	4.625	1.008	20.0	4.00	50	200

^a^ LOD = limit of detection. ^b^ Spanish utilization criteria [193]. ^c^ Spanish landfill criteria [192].
